# Temporal changes of patient characteristics over 12 years in a single-center transcatheter aortic valve implantation cohort

**DOI:** 10.1007/s00392-023-02166-8

**Published:** 2023-02-15

**Authors:** Till Joscha Demal, Jessica Weimann, Francisco Miguel Ojeda, Oliver D. Bhadra, Matthias Linder, Sebastian Ludwig, David Grundmann, Lisa Voigtländer, Lara Waldschmidt, Johannes Schirmer, Niklas Schofer, Stefan Blankenberg, Hermann Reichenspurner, Lenard Conradi, Moritz Seiffert, Andreas Schaefer

**Affiliations:** 1 Department of Cardiovascular Surgery, University Heart & Vascular Center Hamburg, Martinistraße 52, 20246 Hamburg, Germany; 2 Department of Cardiology, University Heart & Vascular Center Hamburg, Martinistraße 52, 20246 Hamburg, Germany

**Keywords:** Guidelines, Low risk, Surgical risk, TAVI, TAVR

## Abstract

**Background:**

Beneficial results of transcatheter aortic valve implantation (TAVI) compared to surgical aortic valve replacement (SAVR) in patients at all risk strata have led to substantial changes in guideline recommendations for valvular heart disease.

**Aim:**

To examine influence of these guideline changes on a real-world TAVI cohort, we evaluated how risk profiles and outcomes of TAVI patients developed in our single-center patient cohort over a period of 12 years.

**Methods:**

Baseline, procedural and 30-day outcome parameters of TAVI patients were retrospectively compared between three time periods (period 1: 2008–2012, period 2: 2013–2017, period 3: 2018–2020).

**Results:**

Between 03/2008 and 12/2020, a total of 3678 patients underwent TAVI at our center. The median age was 81.1 years (25th, 75th percentile: 76.7, 84.9) with no significant change over time. The EuroSCORE II showed a continuous and significant decline from 5.3% (3.3, 8.6) in period 1 to 2.8% (1.7, 5.0) in period 3 (*p < *0.001). Furthermore, rates of permanent pacemaker implantation, acute kidney injury, and paravalvular leakage ≥ moderate continuously declined over time. Accordingly, the 30-day mortality fell from 9.3% in period 1 to 4.3% in period 3 (*p < *0.001).

**Conclusion:**

Despite substantial guideline alterations, median patient age remained largely unchanged in our TAVI cohort over the past 12 years. Therefore, increased age still appears to be the main reason to choose TAVI over SAVR. However, risk profiles declined substantially. Significant improvements in early outcomes suggest favorable influence of less invasive access routes, improved device platforms and growing user experience.

**Graphical abstract:**

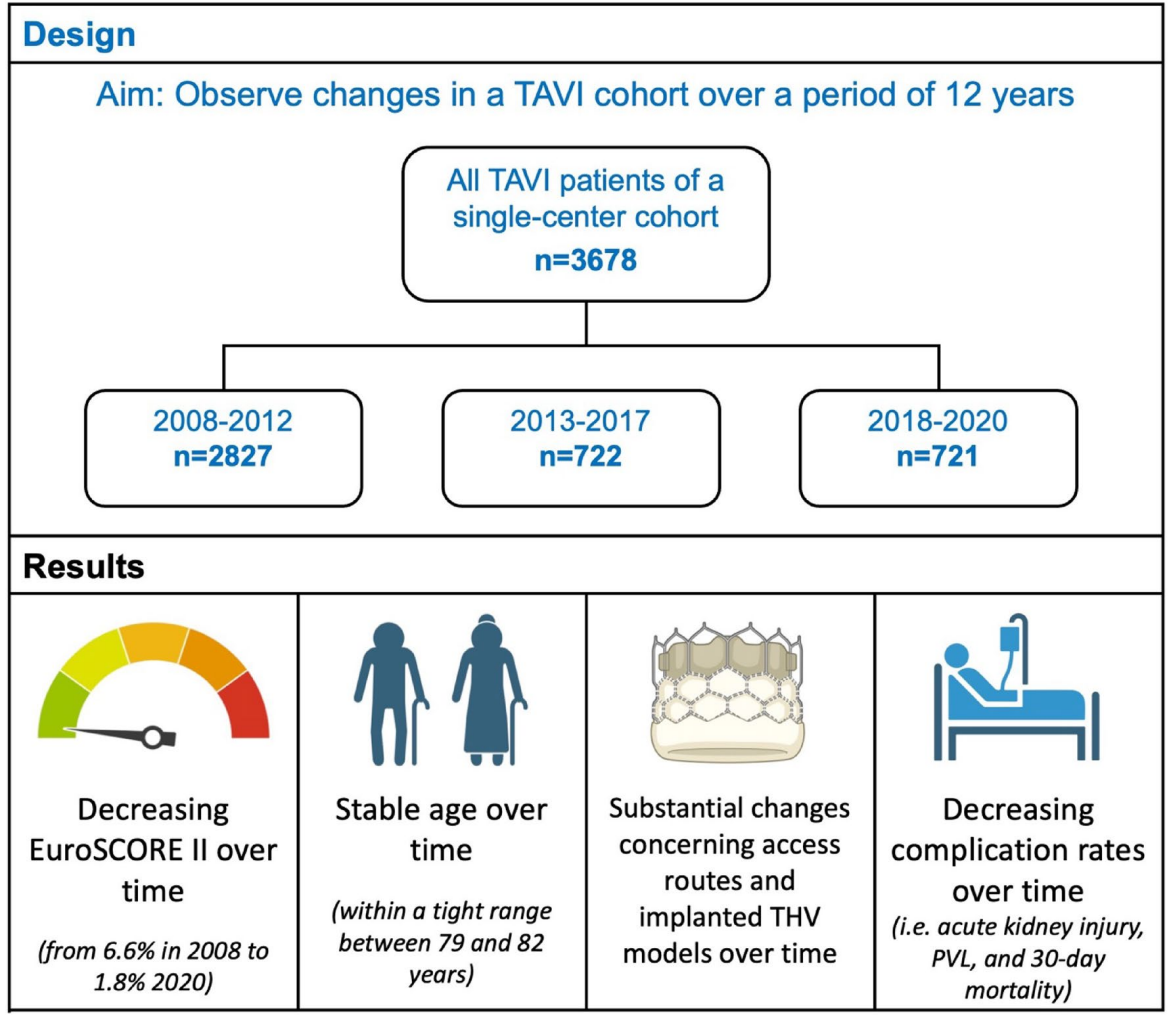

**Supplementary Information:**

The online version contains supplementary material available at 10.1007/s00392-023-02166-8.

## Introduction

Currently, expansion of transcatheter aortic valve implantation (TAVI) indications to patients of younger age and/or lower risk profiles is subject of widespread discussion [1]. While randomized controlled trials (RCT) have shown non-inferiority or even superiority of TAVI over surgical aortic valve replacement (SAVR) in patients at all risk strata [2, 3], concerns regarding residual paravalvular leakage (PVL), postinterventional permanent pacemaker (PPM) implantation, and lack of durability data of transcatheter heart valves (THV) persist. Especially, use of TAVI in younger patients is a subject of controversial discussion since mentioned RCT largely included patients of advanced age. However, these trials resulted in substantial changes of European and North American guidelines [4–9] which have been repeatedly adjusted in recent years. Here, the cut-off values of surgical risk scores from which on TAVI is recommended over SAVR have continuously been lowered [4–9]. To examine the influence of these guideline changes on a real-world TAVI cohort, we evaluated how risk profiles, clinical presentation, and outcomes of TAVI patients developed in our single-center patient cohort over a period of 12 years.

## Materials and methods

### Patients

All patients that underwent TAVI between 03/2008 and 12/2020 at our center were retrospectively registered in a dedicated database.

### Diagnostic work-up and follow-up

Allocation to TAVI followed interdisciplinary consent of a dedicated structural heart team consisting of cardiologists, cardiac surgeons and anesthesiologists. The preprocedural diagnostic work-up followed institutional standards. By routine, all patients received preoperative coronary angiography, transesophageal and/or transthoracic (TTE) echocardiography as well as contrast-enhanced, electrocardiogram-gated multisclice computed tomography. Postprocedural prosthetic valve function was assessed using TTE. Furthermore, patients underwent follow-up TTE in our specialized outpatient clinic for structural heart disease 6 month after implantation and yearly thereafter. In this analysis, we focus on the early outcome of the patient cohort.

### Data acquisition and statistical analysis

Data acquisition was performed anonymized and retrospectively. Therefore, in accordance with German law, no ethical approval is needed and informed patient consent was waived.

To determine changes of the TAVI cohort with time, the total cohort was divided into three different subgroups based on the date of the procedure (period 1: 2008–2012, period 2: 2013–2017, period 3: 2018–2020) to reflect updates of the European guidelines on valvular heart disease in 2012, 2017, and 2021. Baseline, procedural, and outcome parameters were compared between these subgroups. Procedural and 30-day outcomes were adjudicated in accordance with the updated standardized Valve Academic Research Consortium-3 (VARC-3) definitions [10]. Categorical variables were summarized by frequencies and percentages. These were compared between study groups using Chi-squared test. Here, *p* values were computed by Monte Carlo simulation. Continuous variables were described by median and interquartile range (IQR). They were compared between study groups using the Kruskal–Wallis test.

Factors predicting the VARC-3 composite endpoint device success were identified using logistic regression analysis. Factors entered into the model were time period of the procedure, age, gender, EuroSCORE II, access route, choice of THV type, pre-ballooning, post-balloning, valve-in-valve procedure, and the use of cerebral protection devices. Adjusted odds ratios, 95% confidence intervals (CI), and p-values were reported from this model.

The level of significance was set at *α = *0.05 for all analyses. Statistical analyses were computed using R version 4.0.3 statistical software (R Foundation for Statistical Computing, Vienna, Austria; https://www.R-project.org/).

## Results

Between 03/2008 and 12/2020, a total of 3678 patients underwent TAVI at our center. Of these, *n = *722 were assigned to the period 1 group (2008–2012), *n = *1,772 were assigned to the period 2 group (2013–2017), and *n = *1,184 were assigned to the period 3 group (2018–2020).

**Fig. 1:**
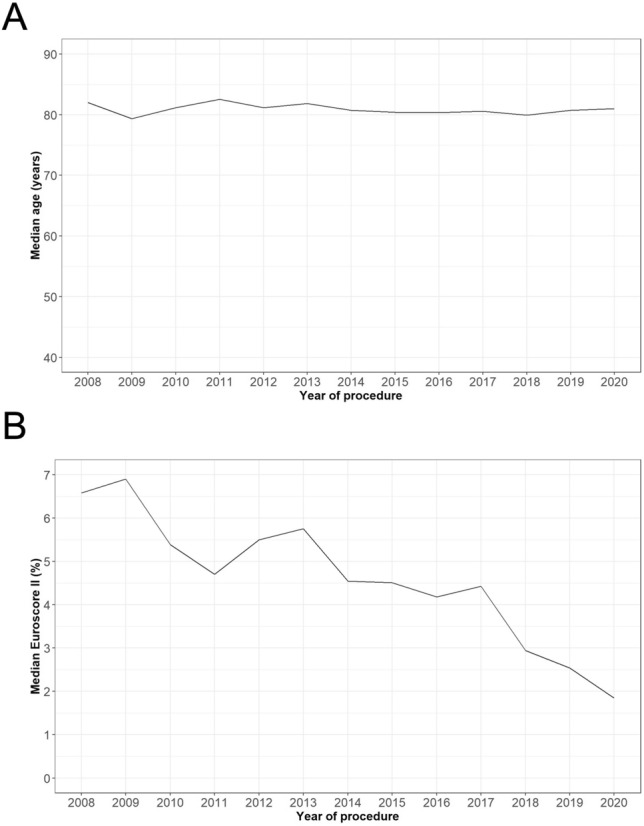
Changes of median age and EuroSCORE II of the transcatheter aortic valve implantation cohort over time. **a** Changes of the median age of the cohort over time. The age of the patients stays stable within a tight range between 79 and 82 years during the whole period of 12 years. **b** Changes of EuroSCORE II of the cohort over time. The EuroSCORE II shows a substantial decline from 6.6% in 2008 to 1.8% in 2020. Supplemental table S1 shows the patient sub cohort with an EuroSCORE II < 4% over all three periods. Here, especially patient risk factors as prior stroke (period 1: 17.4%, period 3: 12.9%; *p = *0.024), chronic lung disease (period 1: 21.5%, period 3: 14.1%; *p = *0.045), and malignant disease (period 1: 36.2%, period 3: 16.2%; *p < *0.001) are statistically significantly higher in the first compared to the last period. Comparing low-risk to all patients in the period 1, the low-risk sub cohort shows a statistically significant higher rate of any malignant disease (36.2% vs. 28.3%, *p = *0.022)

Table 1 shows baseline characteristics of the total cohort and of all three subgroups. Median age was 81.1 years (25th, 75th percentile: 76.7, 84.9). The median age did not significantly change between the three different time periods and stayed within a tight range of 81.0–81.7 (*p = *0.55). This stable patient age is depicted in Fig. 1a. In contrast to age, the EuroSCORE II shows a statistically significant decline from 5.3% (3.3, 8.6) in period 1 to 2.8% (1.7, 5.0) in period 3 (*p < *0.001, Fig. 1b). The same trends for age and EuroSCORE II are found after exclusion of all valve-in-valve patients (Fig. S1). In line with the decreasing EuroSCORE II, the rates of peripheral artery disease, prior stroke, chronic lung disease, malignant disease, and prior cardiac surgery show a significant continuous decline from 2008 to 2020. Furthermore, the rate of at least moderate native aortic valve regurgitation decreased from 24.9% (*n = *170) in the time period 2008–2012 to 14.9% (*n = *165) in the time period 2018–2020 (*p < *0.001).

**Table 1:** Baseline characteristics

	All (*n = *3678)	2008–2012 (*n = *722)	2013–2017 (*n = *1772)	2018–2020 (*n = *1184)	*p* value
Age (years), median (IQR)	81.1 (76.7, 84.9)	81.7 (76.1, 85.3)	81.0 (76.6, 84.8)	81.0 (77.0, 84.7)	0.55
Age (years), range	40, 99	45, 96	46, 96	40, 99	
Male gender, *n* (%)	1879 (51.1)	349 (48.3)	884 (49.9)	646 (54.7)	0.013
EuroSCORE II (%), median (IQR)	4.4 (2.5, 7.7)	5.3 (3.3, 8.6)	4.7 (2.7, 8.0)	2.8 (1.7, 5.0)	< 0.001
Ejection fraction (%), median (IQR)	55.0 (44.0, 60.0)	53.0 (43.0, 60.0)	53.0 (40.0, 60.0)	55.0 (45.0, 60.0)	0.66
Arterial hypertension, *n* (%)	3031 (83.5)	576 (81.1)	1525 (86.3)	930 (80.7)	< 0.001
Diabetes mellitus, *n* (%)	1038 (28.6)	205 (28.9)	520 (29.4)	313 (27.2)	0.42
Coronary artery disease, *n* (%)	2303 (64.4)	454 (63.0)	1148 (65.5)	701 (63.8)	0.46
Peripheral artery disease, *n* (%)	1032 (28.3)	260 (36.0)	541 (30.5)	231 (20.0)	< 0.001
Prior stroke, *n* (%)	564 (15.5)	132 (18.3)	276 (15.6)	156 (13.6)	0.023
Chronic lung disease, *n* (%)	684 (18.8)	188 (26.0)	328 (18.5)	168 (14.5)	< 0.001
Creatinine (mg/dl), median (IQR)	1.1 (0.9, 1.5)	1.1 (0.9, 1.5)	1.1 (0.9, 1.5)	1.1 (0.9, 1.5)	0.33
Any malignant disease, *n* (%)	849 (23.3)	204 (28.3)	430 (24.3)	215 (18.6)	< 0.001
Prior cardiac surgery, *n* (%)	647 (17.8)	168 (23.3)	320 (18.2)	159 (13.9)	< 0.001
Mean transvalvular gradient (mmHg), median (IQR)	33.0 (23.0, 44.9)	33.0 (22.0, 45.0)	32.0 (23.0, 44.7)	33.0 (23.0, 44.0)	0.79
Effective orifice area (cm^2^), median (IQR)	0.8 (0.6, 0.9)	0.7 (0.6, 0.9)	0.8 (0.6, 0.9)	0.8 (0.6, 0.9)	< 0.001
Perimeter derived valve diameter (mm), median (IQR)	24.7 (23.0, 26.4)	24.4 (22.8, 26.4)	24.6 (22.9, 26.3)	25.2 (23.3, 26.6)	0.022
At least moderate aortic regurgitation, *n* (%)	653 (18.8)	170 (24.9)	318 (18.8)	165 (14.9)	< 0.001

Categorical variables were summarized by frequencies and percentages. These were compared between study groups using Chi-squared test. Here, p-values were computed by Monte Carlo simulation. Continuous variables were described by median and interquartile range. They were compared between study groups using the Kruskal–Wallis test

*IQR* interquartile range

Table 2 shows procedural data of the total cohort and of all three subgroups. The rate of transfemoral access shows a substantial and statistically significant increase from 42.0% (*n = *303) in the period 2008–2012 to 95.5% (*n = *1095) in 2018–2020 (*p < *0.001). Correspondingly, the rate of transapical access declined from 57.1% (*n = *412) to 2.4% (*n = *27). According to the fast changing market of THVs and generational changes of THVs, proportions of implanted bioprostheses changed over time. Within the total cohort, Edwards Sapien / Sapien XT/ S3 / S3 Ultra were the most frequently utilized bioprostheses with a rate of 46.2% (*n = *1682) followed by the Boston Scientific Acurate with a rate of 19.7% (*n = *716) and the Medtronic CoreValve / Evolutwith a rate of 15.8% (*n = *576). The rates of pre-balloning significantly decreased whereas the rates of post-balloning increased from period 1 to period 3.

**Table 2:** Procedural data

	All (*N = *3678)	2008–2012 (*N = *722)	2013–2017 (*N = *1772)	2018–2020 (*N = *1184)	*p* value
Access, *n* (%)					< 0.001
Transfemoral	2727 (75.2)	303 (42.0)	1329 (75.6)	1095 (95.5)	
Transapical	810 (22.3)	412 (57.1)	371 (21.1)	27 (2.4)	
Transaxillary	75 (2.1)	6 (0.8)	47 (2.7)	22 (1.9)	
Transaortic	12 (0.3)	1 (0.1)	11 (0.6)	0 (0)	
Other	3 (0.1)	0 (0)	1 (0.1)	2 (0.2)	
Implanted THV, *n* (%)					< 0.001
Edwards Sapien / Sapien XT/ S3 / S3U	1682 (46.2)	468 (64.9)	762 (43.1)	452 (39.4)	
Symetis/Boston Scientific Acurate	716 (19.7)	46 (6.4)	393 (22.2)	277 (24.2)	
Medtronic CoreValve / Evolut / Evolut Pro	576 (15.8)	104 (14.4)	172 (9.7)	300 (26.2)	
JenaValve	182 (5.0)	63 (8.7)	117 (6.6)	2 (0.2)	
SJM Portico	180 (4.9)	0 (0)	114 (6.4)	66 (5.8)	
Boston Scientific Lotus	120 (3.3)	0 (0)	117 (6.6)	3 (0.3)	
Medtronic Engager	95 (2.6)	40 (5.5)	55 (3.1)	0 (0)	
NVT allegra	86 (2.4)	0 (0)	40 (2.3)	46 (4.0)	
Prosthetic valve size (mm), median (IQR)	26.0 (23.0, 27.0)	26.0 (23.0, 26.0)	26.0 (23.0, 27.0)	26.0 (25.0, 29.0)	< 0.001
Valve-in-Valve procedure, *n* (%)	193 (5.4)	23 (3.2)	90 (5.1)	80 (7.4)	< 0.001
Pre-ballooning, *n* (%)	2682 (74.3)	597 (82.7)	1224 (69.5)	861 (76.3)	< 0.001
Post-ballooning, *n* (%)	1198 (33.4)	111 (15.6)	590 (33.6)	497 (44.3)	< 0.001
Contrast agent (ml), median (IQR)	165.0 (122.0, 214.0)	150.0 (113.2, 205.0)	160.0 (116.0, 210.0)	180.0 (142.8, 221.0)	< 0.001
Use of cerebral protection device, *n* (%)	427 (11.8)	0 (0)	257 (14.6)	170 (15.0)	< 0.001

Categorical variables were summarized by frequencies and percentages. These were compared between study groups using Chi-squared test. Here, p-values were computed by Monte Carlo simulation. Continuous variables were described by median and interquartile range. They were compared between study groups using the Kruskal–Wallis test

*IQR* interquartile range, *NVT* new valve technology, *SJM* St. Jude Medical, *THV* transcatheter heart valve

**Fig. 2:**
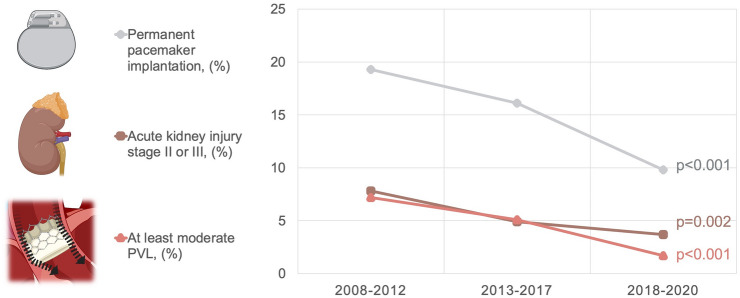
Rates of the postprocedural complications permanent pacemaker implantation, acute kidney injury, and PVL ≥ moderate over the three different analyzed time periods. All three depicted complication rates show a continuous decrease over time. Created using BioRender.com. *PVL* paravalvular leakage

Table 3 shows the early outcome of the total cohort and the three subgroups. All observed complication rates did either significantly decrease or remained stable over time. Major vascular complications occurred in 6.6% (*n = *239) of the total cohort with no significant change of this rate over time (*p = *0.15). Type 3 or 4 bleeding was found in 12.4% (*n = *451) of the patients. The bleeding rate increased from 11.1% (*n = *80) in period 1 to 14.1% (*n = *248) in period 2 and significantly fell to 10.6% (*n = *123) in period 3 (*p = *0.013). As depicted in Fig. 2, rates of PPM, acute kidney injury stage II or III, and PVL ≥ moderate showed a statistically significant and continuous decline over time. Most pronounced was the decrease of PVL ≥ moderate from 7.2% (*n = *46) in 2008–2012 to 1.7% (*n = *1.7%) in 2018–2020. Within the total cohort, rates of myocardial infarction and stroke with disability were 1.1% (*n = *41) and 3.6% (*n = *130), respectively, and did not show statistically significant changes over time. The overall 30-day mortality was 6.2% (*n = *229) and showed a statistically significant decline from 9.3% (*n = *67) in 2008–2012 to 4.3% (*n = *51) in 2018–2020 (*p < *0.001).

**Table 3:** Early outcome

	All (*N = *3678)	2008–2012 (*N = *722)	2013–2017 (*N = *1772)	2018–2020 (*N = *1184)	*p* value
Valve malposition, *n* (%)	79 (2.5)	17 (2.4)	50 (2.8)	12 (1.7)	0.29
Pericardial tamponade, *n* (%)	20 (0.6)	4 (0.6)	8 (0.5)	8 (1.2)	0.14
Coronary ostia occlusion, *n* (%)	14 (0.4)	4 (0.6)	7 (0.4)	3 (0.4)	0.94
Aortic root rupture, *n* (%)	11 (0.3)	2 (0.3)	5 (0.3)	4 (0.6)	0.56
Conversion to CPB, *n* (%)	63 (1.8)	18 (2.5)	31 (1.8)	14 (1.4)	0.20
Length of ICU stay (days), median (IQR)	1.0 (1.0, 2.0)	1.0 (1.0, 3.0)	1.0 (1.0, 3.0)	1.0 (1.0, 2.0)	< 0.001
Major vascular complication, *n* (%)	239 (6.6)	54 (7.5)	122 (6.9)	63 (5.4)	0.15
Type 3 or 4 bleeding, *n* (%)	451 (12.4)	80 (11.1)	248 (14.1)	123 (10.6)	0.013
Permanent pacemaker implantation, *n* (%)	535 (14.7)	139 (19.3)	283 (16.1)	113 (9.8)	< 0.001
Acute kidney injury stage II or III, *n* (%)	186 (5.1)	56 (7.8)	87 (4.9)	43 (3.7)	0.002
Myocardial infarction, *n* (%)	41 (1.1)	9 (1.2)	25 (1.4)	7 (0.6)	0.11
Disabling stroke, *n* (%)	130 (3.6)	25 (3.5)	68 (3.8)	37 (3.2)	0.62
Postprocedural mean gradient (mmHg), median (IQR)	9.0 (6.0, 12.0)	9.0 (6.0, 12.0)	9.0 (6.4, 13.0)	8.0 (5.0, 11.0)	< 0.001
At least moderate PVL, *n* (%)	148 (4.4)	46 (7.2)	83 (5.1)	19 (1.7)	< 0.001
VARC Device Success, *n* (%)	3374 (92.4)	632 (87.5)	1634 (92.4)	1108 (95.4)	< 0.001
30-day mortality, *n* (%)	229 (6.2)	67 (9.3)	111 (6.3)	51 (4.3)	< 0.001

Categorical variables were summarized by frequencies and percentages. These were compared between study groups using Chi-squared test. Here, p-values were computed by Monte Carlo simulation. Continuous variables were described by median and interquartile range. They were compared between study groups using the Kruskal–Wallis test

*CPB* cardiopulmonary bypass, *ICU* intensive care unit, *IQR* interquartile range, *PVL* paravalvular leakage

**Fig. 3:**
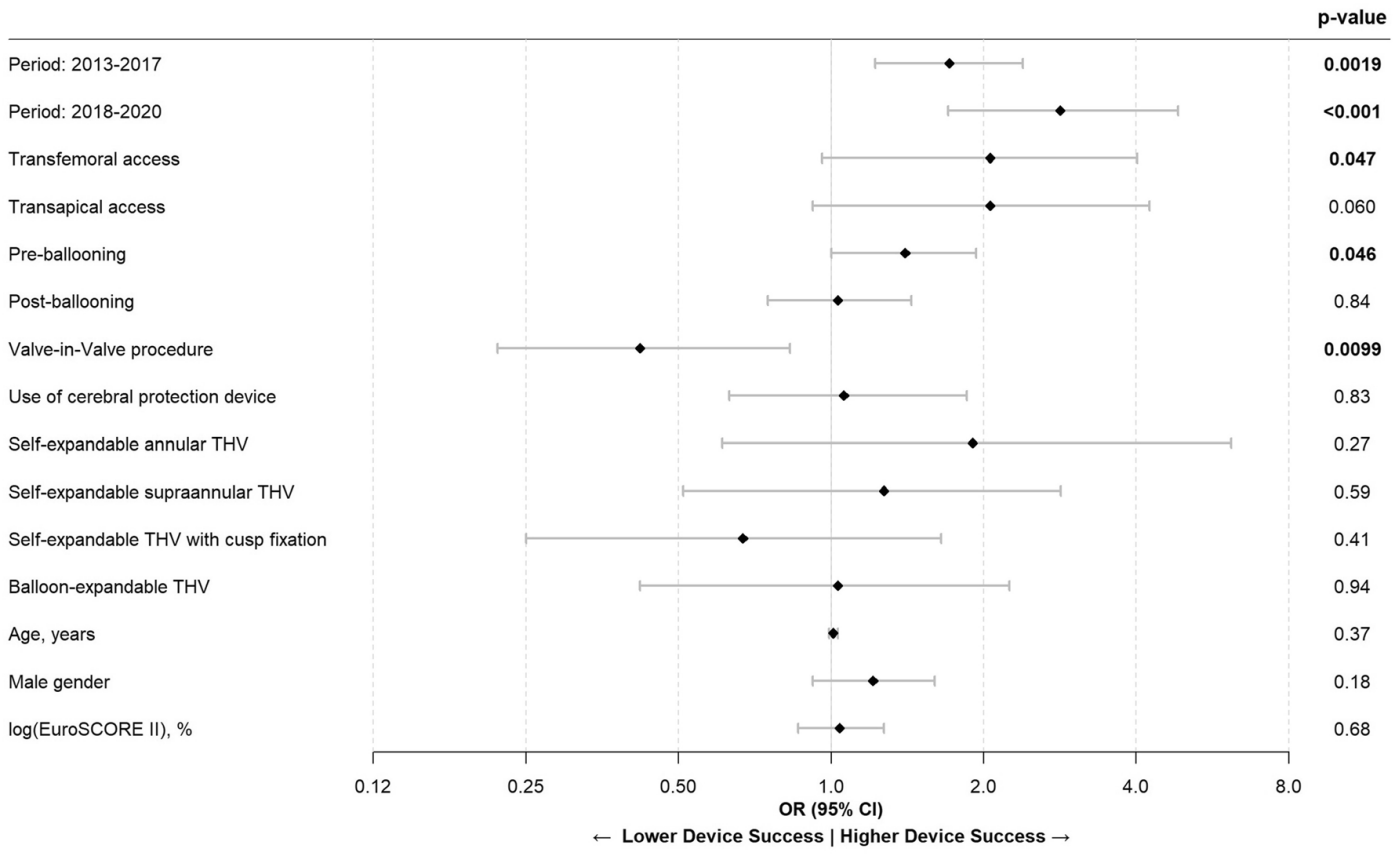
Forest plot of the multivariate regression model used to identify factors predicting the VARC-3 composite endpoint device success. Odds ratios and 95% confidence intervals of all tested parameters in the model are shown using a logarithmic x-axis. Independent predicting factors identified in the regression analysis were the more recent time periods 2013–2017 and 2018–2020 (when compared to the period 2008–2012), as well as transfemoral access in comparison to other access routes, and pre-ballooning. Neither THV choice nor age and EuroSCORE II did independently predict device success. THV: Transcatheter heart valve. VARC-3: Valve academic research consortium 3

The VARC-3 endpoint device success is a composite of technical success, freedom from mortality, freedom from surgery or intervention related to the device or to a major vascular or access-related or cardiac structural complication. The rate of device success increased over time from 87.5% (*n = *632) in period 1 to 95.4% (*n = *1108) in period 3 which was mainly driven by falling rates of PVL and decreasing mortality. To identify factors predicting VARC-3 device success, we performed a multivariate logistic regression analysis. The resulting forest plot with all parameters included in the model is shown in Fig. 3. Independent predicting factors identified in the regression analysis were the more recent time periods (period 2013–2017: odds ratio (OR) 1.71 (95% CI 1.22, 2.39), *p = *0.0019; period 2018–2020: OR 2.83 (95% CI 1.70, 4.84), *p < *0.001), transfemoral access (OR 2.06 (95% CI 0.96, 4.02), *p = *0.047), and pre-ballooning (OR 1.40 (95% CI 1.00, 1.93), *p = *0.046). The THV choice did not independently predict device success.

To reassure that the improved results over time are not only due to declining risk profiles of the patients, we stratified the outcome analysis by EuroSCORE II and analyzed the outcome of a sub cohort of low-risk patients (EuroSCORE II < 4%). A total of 1,366 patients had a EuroSCORE II < 4%. Similar to the multivariate regression model results, the VARC device success rates improved over time in this sub cohort as well (period 1: 83.8%, period 2: 91.9%, period 3: 96.5%, *p < *0.001).

Table 4 shows transthoracic follow-up echocardiography of the cohort 12 months after implantation. A total of 422 patients were available for follow-up echocardiography. The mean transvalvular gradient significantly decreased over time (period 1: 10.0 mmHg, period 2: 10.0 mmHg, period 3: 8.0 mmHg; *p = *0.009). Accordingly, moderate PVL rates tended to decrease (period 1: 10.2%, period 2: 8.3%, period 3: 0.0%; *p = *0.099) and the rate of patients with no PVL tended to increase (period 1: 42.9%, period 2: 47.7%, period 3: 62.5%; *p = *0.069). Furthermore, the rate of patients with no transvalvular regurgitation increased over time (period 1: 87.6%, period 2: 93.9%, period 3: 100.0%; *p = *0.017).

**Table 4:** Follow-up transthoracic echocardiography 12 months after implantation

	All (*n = *422)	2008–2012 (*n = *282)	2013–2017 (*n = *133)	2018–2020 (*n = *40)	*p* value
Mean transvalvular gradient (mmHg), median (IQR)	10.0 (7.0, 13.0)	10.0 (7.8, 14.0)	10.0 (7.0, 13.0)	8.0 (6.0, 10.0)	0.009
Paravalvular regurgitation, *n* (%)					
None	206 (46.1)	118 (42.9)	63 (47.7)	25 (62.5)	0.069
Trace	37 (8.3)	19 (6.9)	18 (13.6)	0 (0)	0.009
Mild	163 (36.5)	108 (39.3)	40 (30.3)	15 (37.5)	0.21
Moderate	39 (8.7)	28 (10.2)	11 (8.3)	0 (0)	0.099
Severe	2 (0.4)	2 (0.7)	0 (0)	0 (0)	0.63
Transvalvular regurgitation, *n* (%)					
None	405 (90.6)	241 (87.6)	124 (93.9)	40 (100)	0.017
Trace	7 (1.6)	1 (0.4)	6 (4.5)	0 (0)	0.012
Mild	31 (6.9)	30 (10.9)	1 (0.8)	0 (0)	0.001
Moderate	4 (0.9)	3 (1.1)	1 (0.8)	0 (0)	1.00
Severe	0 (0)	0 (0)	0 (0)	0 (0)	-
Left ventricular ejection fraction, *n* (%)					
≥ 55%	315 (69.2)	197 (69.9)	93 (69.9)	25 (62.5)	0.65
45–54%	80 (17.6)	55 (19.5)	19 (14.3)	6 (15.0)	0.37
30–44%	42 (9.2)	22 (7.8)	11 (8.3)	9 (22.5)	0.012
< 30%	18 (4.0)	8 (2.8)	10 (7.5)	0 (0)	0.027

Categorical variables were summarized by frequencies and percentages. These were compared between study groups using Chi-squared test. Here, *p* values were computed by Monte Carlo simulation. Continuous variables were described by median and interquartile range. They were compared between study groups using the Kruskal–Wallis test

*IQR* interquartile range

## Discussion

Main findings of this study are:

1. The median EuroSCORE II of the TAVI cohort showed a distinct decline within the analyzed 12 years from 6.6% in 2008 to 1.8% in 2020.
2. In contrast to the EuroSCORE II, median age of the TAVI cohort remained stable during the analyzed time period of 12 years within a tight range between 79 and 82 years*.*
3. Within the analyzed 12 years, substantial procedural changes were found concerning the choice of the access routes and the implanted THV models.
4. Complication rates decreased over time. Especially, rates of PPM, acute kidney injury, and PVL ≥ moderate showed a significant and continuous decline. Accordingly, the 30-day mortality fell from 9.3% in 2008–2012 to 4.3% in 2018–2020 (*p < *0.001).
5. In line with declining complication rates, the VARC-3 device success endpoint was reached with increasing rates over time. Logistic regression analysis revealed more recent time periods, transfemoral access, and pre-ballooning to be independent predictors for device success.
6. Characterized by decreasing rates of moderate regurgitation and increasing rates of patients with no regurgitation, follow-up echocardiography revealed improved hemodynamic results after 1-year over the three reported periods.

### EuroSCORE II and age

In line with the growing evidence that TAVI is non-inferior or beneficial compared to SAVR in intermediate- and low-risk cohorts [11–14], our TAVI cohort showed a decline of the surgical risk represented by the EuroSCORE II. This reduced EuroSCORE II is mainly triggered by decreasing rates of peripheral artery disease, chronic lung disease, and prior cardiac surgery.

In the ESC/EACTS guidelines on valvular heart disease of 2012, TAVI was only recommended in patients with severe symptomatic aortic stenosis who were not suitable for SAVR or had a high surgical risk with an estimated early mortality risk of > 10% [5]. The 2014 AHA/ACC guidelines on valvular heart disease already stated a lower cut-off of 8% [8]. The current European Guidelines of 2021 recommend SAVR in patients with a EuroSCORE II < 4% and TAVI in patients with a EuroSCORE II > 8% [4], whereas the North American guidelines of 2020 stick with the cut-off of 8% [7]. However, when compared to these guideline recommendations, our TAVI cohort shows a rather low-risk profile during all three analyzed time periods. To further evaluate the rather low-risk profile of our cohort we analyzed the baseline characteristics of the sub cohort of patients with an EuroSCORE II < 4%. In comparison to the total cohort of period 1, low-risk patients in period 1 presented with a statistically significant higher rate of any malignant disease (36.2% vs. 28.3%, *p = *0.022). Therefore, this and other factors not included in the EuroSCORE II may have contributed to the choice of TAVI over SAVR in this sub cohort. Other factors that substantially influence our decision to allocate patients to TAVI in the clinical routine include frailty, porcelain aorta, sequelae of chest radiation, high likelihood of patient-prosthesis mismatch, suitable anatomy for TAVI, no other indication for open heart surgery, and severe chest deformation. Nevertheless, as the median age of 81.1 years stayed relatively stable over all three time periods, age appears to be the main trigger to choose TAVI over SAVR in the analyzed cohort.

Contrarily, the favorable results of the TAVI procedure presented in the PARTNER 3 and the Evolut Low Risk study [13, 14] spark the discussion on the use of transcatheter aortic valve replacement in low-risk patients of younger age. However, so far TAVI has not been systematically tested in young patients (< 65 years). While the early randomized TAVI studies only included elderly patients with a mean age over 80 years, the mean age in the low-risk studies ranged between 73 and 74 years. Outcome data on the use of THVs in patients below the age of 70 have only been generated in patients with particularly high surgical risk due to severe comorbidities and cannot be transferred to a low-risk patient group [1]. The eagerly awaited results from the NOTION-2 trial (NCT02825134), which recruited patients with severe aortic stenosis below 75 years of age and randomized between TAVI and SAVR will therefore be of great importance to clarify this issue.

When discussing TAVI in younger patients, THV durability is a major issue. So far, only scarce long-term data on the use of THVs exist. Results from the NOTION trial comparing the outcome of SAVR vs. TAVI in a low-risk cohort did not show a statistically significant difference in the rate of bioprosthetic valve failure after 8 years [15]. However, in the SAVR arm, a total of 34% received Sorin Mitroflow or SJM Trifecta prostheses, which both are models with a known high rate of early degeneration [16, 17]. Generally, studies with a follow-up of 5–10 years show a comparatively high rate of moderate to severe structural valve degeneration of 8.7–9.1% after TAVI [15, 18]. These results appear particularly surprising as the analyzed patient cohort is of relatively high mean age (79.3 years), since early valve degeneration after surgical aortic valve replacement is known to be more likely in patients under the age of 50 [19]. In contrast, when using the Perimount prosthesis (Edwards Lifesciences Inc., Irvine, CA, US), SAVR is associated with excellent long-term results. Here, the freedom from structural valve degeneration is 100% after 5 years and 98.1% after 10 years [17]. However, currently different techniques are discussed to optimize TAVI outcomes especially in younger patients. These techniques include implantation of self-expandable THV using the cusp-overlap technique to reduce the need for postprocedural PPM, commissural alignment of bioprostheses to obtain optimal coronary access in patients with pre-existing coronary artery disease, and sophisticated valve size choice to reduce possible hypoattenuated leaflet thickening and/or restricted leaflet motion. The herein shown age distribution over all three time episodes indicates, that currently the described techniques may be suitable for only a minority of TAVI patients. Nevertheless, these procedures are of utmost importance and are performed in high frequency in selected patients at our center.

Especially at the start of the TAVI program at our center (period 1), age indeed was the main reason to choose TAVI over SAVR in clinical discussions. Today, decision making became more complex and other factors like comorbidities, frailty, anticipated hemodynamic outcome, and lifetime management gained importance. Due to the higher rates of paravalvular leakages and pacemaker implantations and the lacking data on the long-term durability of THVs, we rarely perform TAVI in patients younger than 75 years. In these young patients, the main triggers for TAVI are severe comorbidities, frailty, porcelain aorta, and a high likelihood of patient-prosthesis mismatch. However, due to the promising results of the low-risk TAVI trials, we expect our cohort to become younger soon resulting in a mean age of 75 to 80 years.

### Choice of access route

Within the analyzed time period of 12 years, substantial changes were found concerning the choice of the access route and of the implanted THVs. The rate of transfemoral access showed a substantial and statistically significant increase, whereas the rate of transapical access continuously declined from 2008 to 2020. Transapical access is well-known to be associated with a higher early mortality, higher rates of bleeding, and acute kidney injury as well as longer in-hospital stays when compared to the transfemoral approach [20]. However, traditionally patients provided with transapical TAVI presented a significant higher comorbidity burden compared to patients undergoing the transfemoral approach [21]. On the other hand, rates of transfemoral TAVI were increased by growing experience with pre-treatment techniques of iliac vessels, e.g. percutaneous transluminal angioplasty, intravasal lithoplasty (Shockwave IVL, Shockwave Medical, Inc.), or even placement of stents prior to TAVI.

### Postprocedural complications: PPM and PVL

The rate of PPM implantations after TAVI continuously decreased over time in this analysis. Due to subsequent complications such as device-associated endocarditis, lead fracture, lead perforation, and tricuspid valve regurgitation, the need for a PPM after TAVI is associated with reduced long-term survival and should therefore be avoided [22]. Among known risk factors for PPM implantation after TAVI are low valve implantation depth, membranous septum length, prior right bundle branch block, use of self-expandable valves, and high EuroSCORE II [23, 24]. Therefore, the declining PPM rates may be partially explained by the over time decreasing EuroSCORE II of our cohort and the growing experience regarding implantation depth with self-expandable THV.

Furthermore, over time declining rates of PVL could be detected early after implantation and in the 1-year follow-up echocardiography of the analyzed cohort. As PVL is known to be associated with a dramatic increase in 1-year mortality after TAVI, measures to prevent PVL are mandatory [25]. Known independent predictors of PVL are the use of self-expanding THVs, the presence of left ventricular outflow tract calcification, high Agatston scores, and THV malpositioning [26, 27]. Calcification patterns and Agatson scores were not examined in this study. As the use of self-expandable THVs increased over time in our cohort, this factor does not explain the falling rate of PVL. However, growing user experience regarding optimal implantation depth and handling of different calcification patterns as well as modern device platforms with outer skirts may have played an important role in preventing PVL in recent years.

### Device success

Within the 12-year time span, the VARC-3 composite endpoint device success was reached with increasing rates over time, which was mainly driven by falling rates of PVL and decreasing mortality. Multivariate regression analysis revealed the more recent time periods, transfemoral access, and pre-ballooning to be independent predictors for device success. The beneficial results in recent years regarding the VARC device success endpoint may imply a relevant impact of the growing user experience and of improved device platforms.

## Conclusion

Reflected by frequencies of relevant comorbidities and EuroSCORE II, risk profiles changed dramatically towards lower risk in our TAVI cohort over the past 12 years although median patient age remained largely unchanged. In conclusion, despite profound changes in guideline recommendations for treatment of valvular heart disease, increased age still appears to be the main reason to choose TAVI over SAVR at our center. However, allocation to TAVI over SAVR should always be discussed in an interdisciplinary team considering a variety of individual patient characteristics (age, comorbidities, frailty, porcelain aorta, sequelae of chest radiation, likelihood of patient-prosthesis mismatch, suitability of the anatomy for TAVI, other indications for open heart surgery despite aortic valve disease, severe chest deformation etc.). Furthermore, our analysis showed significant improvements in early outcomes suggesting favorable influence of less invasive access routes, improved device platforms and growing user experience over a period of 12 years.

### Impact on daily practice

The EuroSCORE II showed a continuous and significant decline with accompanying significant decrease of frequencies of major comorbidities over time. The extension of TAVI treatment to a cohort of lower risk underlines the importance of the interdisciplinary heart team in which an in-depth discussion of every patient with aortic valve disease enables an optimal individualized treatment.

The median age of our cohort was 81.1 years and stayed relatively stable over all three time periods. Therefore, our results suggest that certain contemporary implantation techniques like use of cusp overlap projections and commissural alignment, which are of particular importance in younger patients, may be suitable for only a minority of patients currently provided with TAVI.

## Limitations

The main limitation of our study is its retrospective nature. The single-center design leads to the presentation of a specific approach in treatment allocation of patients with aortic valve disease. However, due to the high number of patients in our cohort, main findings as decreasing risk and procedural changes may well represent patient care in industrialized nations. For further insights, multicenter analyses are needed.

## Supplementary Information

Below is the link to the electronic supplementary material.

Supplementary file1 (DOCX 372 kb)

## Data Availability

Due to German data protection laws, data sharing will only be possible after approval by the ethics committee of the medical chamber Hamburg. For data request, please contact the corresponding author.
