# Supplementary material for: Temporal changes of patient characteristics over 12 years in a single-center transcatheter aortic valve implantation cohort
Source: Clin Res Cardiol. 2023 Feb 15;112(5):691–701. doi: 10.1007/s00392-023-02166-8 (PMC10160189; doi:10.1007/s00392-023-02166-8)
Supplement: Supplementary file 1 — Supplementary file1 (DOCX 372 kb) [file 392_2023_2166_MOESM1_ESM.docx]

Temporal changes of patient characteristics over 12 years in a single-center transcatheter aortic valve implantation cohort

**Till Joscha Demal^1^ • Jessica Weimann^2^ • Francisco Miguel Ojeda^2^ • Oliver D. Bhadra^1^ • Matthias Linder^2^ • Sebastian Ludwig^2^ • David Grundmann^2^ • Lisa Voigtländer^2^ • Lara Waldschmidt^2^ • Johannes Schirmer^1^ • Niklas Schofer^2^ • Stefan Blankenberg^2^ • Hermann Reichenspurner^1^ • Lenard Conradi^1^ • Moritz Seiffert^2^ • Andreas Schaefer^1^**

^1^ Department of Cardiovascular Surgery, University Heart & Vascular Center Hamburg, Martinistraße 52, D-20246 Hamburg, Germany

^2^ Department of Cardiology, University Heart & Vascular Center Hamburg, D-20246 Hamburg, Germany

- Till Joscha Demal
  t.demal@uke.de

**SUPPLEMENTARY FIGURES**

**
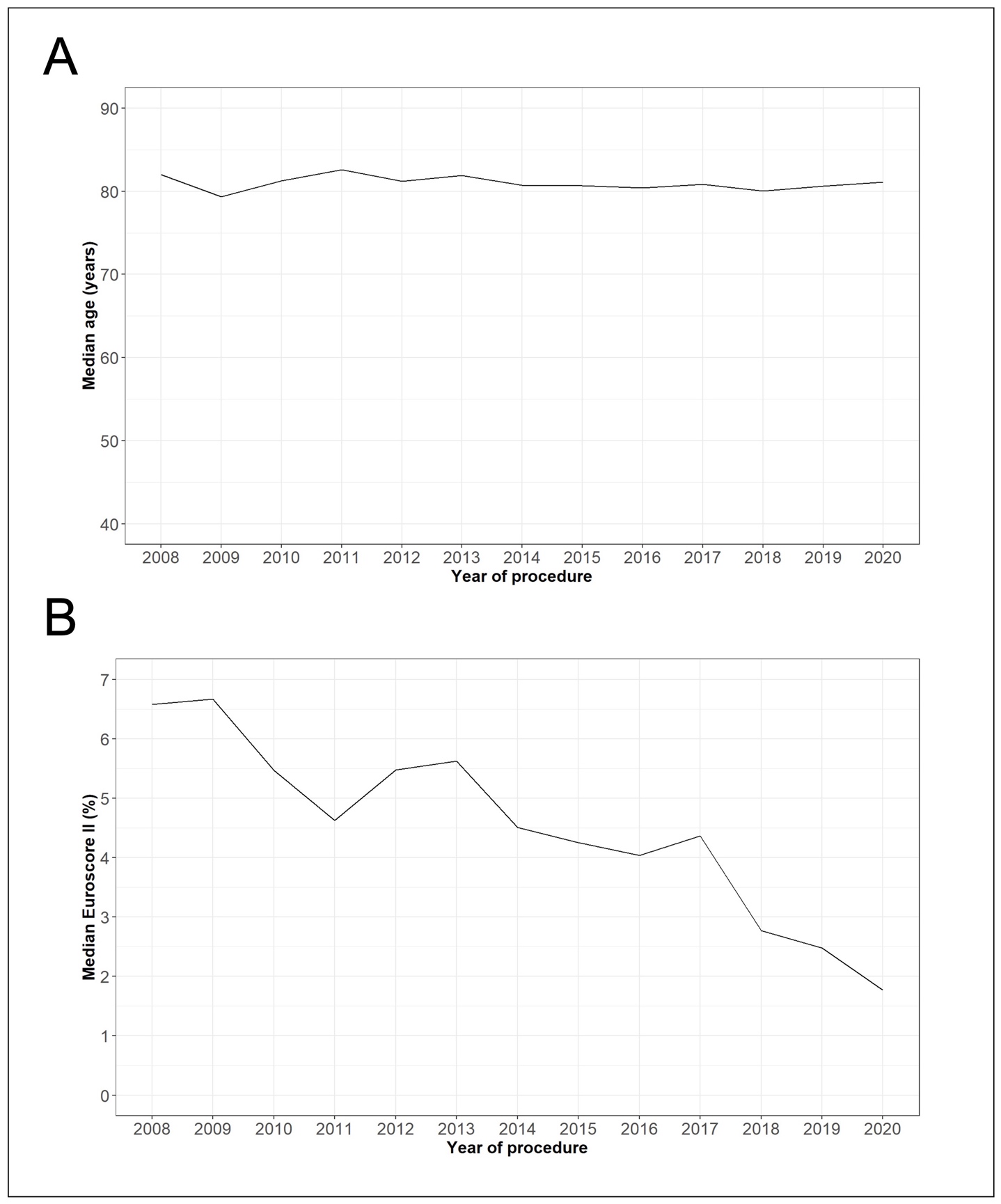
Figure S1: Changes of median age and EuroSCORE II of the transcatheter aortic valve implantation cohort after exclusion of Valve-in-Valve patients over time.** A: Changes of the median age of the cohort over time. The age of the patients stays stable within a tight range during the whole period of 12 years. B: Changes of EuroSCORE II of the cohort over time. The EuroSCORE II shows a strong decline over time.

**Table S1** Baseline characteristics of sub cohort with EuroSCORE II < 4%

|  | All  (N=1366) | 2008-2012  (N=247) | 2013-2017  (N=692) | 2018-2020  (N=427) | p-value |
| --- | --- | --- | --- | --- | --- |
| Age (years), median (IQR) | 80.1 (75.6, 83.7) | 80.2 (74.5, 83.8) | 79.9 (75.4, 83.5) | 80.6 (76.2, 84.0) | 0.052 |
| Male Gender, n (%) | 709 (51.9) | 115 (46.6) | 365 (52.7) | 229 (53.6) | 0.17 |
| EuroSCORE II, median (IQR) | 2.4 (1.8, 3.1) | 2.8 (1.9, 3.4) | 2.5 (1.9, 3.1) | 2.0 (1.5, 2.8) | **<0.001** |
| Ejection fraction (%), median (IQR) | 55.0 (50.0, 60.0) | 60.0 (50.8, 60.2) | 60.0 (55.0, 63.0) | 55.0 (50.0, 60.0) | **0.036** |
| Arterial hypertension, n (%) | 1135 (83.1) | 197 (79.8) | 582 (84.1) | 356 (83.4) | 0.30 |
| Diabetes mellitus, n (%) | 344 (25.2) | 59 (23.9) | 176 (25.4) | 109 (25.6) | 0.88 |
| Coronary artery disease, n (%) | 763 (56.4) | 126 (51.0) | 395 (57.7) | 242 (57.3) | 0.17 |
| Peripheral arterial disease, n (%) | 246 (18.0) | 50 (20.2) | 128 (18.5) | 68 (16.0) | 0.35 |
| Prior stroke, n (%) | 172 (12.6) | 43 (17.4) | 74 (10.7) | 55 (12.9) | **0.024** |
| Chronic lung disease, n (%) | 226 (16.5) | 53 (21.5) | 113 (16.3) | 60 (14.1) | **0.045** |
| Creatinine (mg/dl), median (IQR) | 1.0 (0.8, 1.2) | 1.0 (0.8, 1.2) | 0.9 (0.8, 1.1) | 1.0 (0.9, 1.3) | **<0.001** |
| Any malignant disease, n (%) | 339 (24.9) | 89 (36.2) | 181 (26.2) | 69 (16.2) | **<0.001** |
| Prior cardiac surgery, n (%) | 42 (3.1) | 7 (2.8) | 21 (3.0) | 14 (3.3) | 0.96 |
| Mean transvalvular gradient (mmHg), median (IQR) | 36.0 (27.0, 47.0) | 37.0 (27.0, 48.0) | 37.0 (27.3, 48.0) | 35.0 (26.0, 46.0) | 0.17 |
| Effective orifice area (cm^2^), median (IQR) | 0.8 (0.6, 0.9) | 0.7 (0.6, 0.9) | 0.8 (0.7, 0.9) | 0.8 (0.6, 0.9) | **<0.001** |
| Perimeter derived valve diameter (mm), median (IQR) | 24.9 (23.1, 26.5) | 24.8 (23.0, 26.3) | 24.7 (23.0, 26.5) | 25.2 (23.2, 26.5) | 0.43 |
| At least moderate aortic regurgitation, n (%) | 230 (17.5) | 67 (28.8) | 98 (14.7) | 65 (15.7) | **<0.001** |

Categorical variables were summarized by frequencies and percentages. These were compared between study groups using Chi-squared test. Here, p-values were computed by Monte Carlo simulation. Continuous variables were described by median and interquartile range. They were compared between study groups using the Kruskal-Wallis test. IQR: Interquartile range.

|  | **Patients <75 years**,  n = 685 | **Patients ≥75 years**,  n = 2,991 | **p-value** |
| --- | --- | --- | --- |
| **EuroSCORE II (%),** median (IQR) | 3.4 (1.9, 6.8) | 4.5 (2.7, 7.9) | <0.001 |
| The p-value was computed using the Wilcoxon rank sum test. IQR: Interquartile range. | | | |

**Table S2** EuroSCORE II in patients below and over 75 years

**Table S3** EuroSCORE II in patients below 75 years over the three periods

|  | **2008-2012**,  n = 148 | **2013-2017**,  n = 328 | **2018-2020**,  n = 209 | **p-value** |
| --- | --- | --- | --- | --- |
| **EuroSCORE II (%),** median (IQR) | 4.5 (2.1, 8.7) | 3.8 (2.1, 6.7) | 2.4 (1.3, 4.8) | <0.001 |
| The p-value was computed using the Kruskal-Wallis rank sum test. IQR: Interquartile range. | | | | |

**Table S4** EuroSCORE II in patients ≥75 years over the three periods

|  | **2008-2012**,  n = 573 | **2013-2017**,  n = 1,443 | **2018-2020**,  n = 975 | **p-value** |
| --- | --- | --- | --- | --- |
| **EuroSCORE II (%),** median (IQR) | 5.5 (3.5, 8.6) | 4.9 (2.9, 8.3) | 2.9 (1.8, 5.1) | <0.001 |
| The p-value was computed using the Kruskal-Wallis rank sum test. IQR: Interquartile range. | | | | |

**Table S5** Multivariate regression model predicting 30-day mortality

|  | OR (95% CI) | p-value |
| --- | --- | --- |
| Transfemoral access | 0.56 (0.26, 1.19) | 0.13 |
| Ejection fraction (%) | 0.97 (0.95, 0.99) | 0.011 |
| Coronary artery disease | 1.45 (0.79, 2.67) | 0.23 |
| Prior stroke | 0.48 (0.12, 1.88) | 0.29 |
| Chronic lung disease | 1.60 (0.74, 3.44) | 0.23 |
| Creatinine (mg/dl) | 1.08 (0.80, 1.44) | 0.63 |
| Any malignant disease | 0.59 (0.26, 1.36) | 0.22 |
| Prior cardiac surgery | 1.72 (0.80, 3.72) | 0.17 |
| Age (years) | 1.04 (0.98, 1.11) | 0.18 |
| Male Gender | 0.96 (0.52, 1.76) | 0.88 |

To evaluate possible risk factor for early mortality, we performed a multivariate regression model to predict 30-day mortality. Only ejection fraction was identified as predictor for 30-day mortality.
